# Research hotspots and trends of neuroimaging in social anxiety: a CiteSpace bibliometric analysis based on Web of Science and Scopus database

**DOI:** 10.3389/fnbeh.2024.1448412

**Published:** 2024-12-05

**Authors:** Peng Zhang, Jianing Zhang, Mingliang Wang, Shuyuan Feng, Yuqing Yuan, Lin Ding

**Affiliations:** ^1^Key Research Base of Humanities and Social Sciences of the Ministry of Education, Academy of Psychology and Behavior, Tianjin Normal University, Tianjin, China; ^2^Faculty of Psychology, Tianjin Normal University, Tianjin, China

**Keywords:** social anxiety, brain mechanism, knowledge map, visual analysis, CiteSpace

## Abstract

**Background:**

This study focused on the research hotspots and development trends of the neuroimaging of social anxiety (SA) in the past 25 years.

**Methods:**

We selected 1,305 studies on SA neuroimaging from the Web of Science and Scopus from January 1998 to December 2023. CiteSpace was used to analyze the number of published articles visually, cited references, cooperation among authors and institutions, co-occurrence of keywords, clustering of keywords, burst of keywords, and time zone of co-occurring keywords.

**Results:**

A total of 1,305 articles were included, and the annual number of articles published over nearly 25 years showed the overall trend is on the rise. The analysis of author and institutional collaboration reveals that most authors collaborate closely. Among them, the team led by Pine, Daniel S published 59 articles, making it the most central team. Harvard University is identified as the most central institution in this network. The research hotspots can be categorized into four areas: research techniques, cognitive processing research areas, core brain regions and brain networks, and the neural predictors of treatment outcomes in SA. The most recent burst keywords are “cognitive behavioral therapy,” “systematic review,” “machine learning,” “major clinical study,” “transcranial direct current stimulation,” “depression,” and “outcome assessment,” which provided clues on research frontiers. Based on the burst map and keyword time zone map, it appears that exploring the activity of brain regions involved in cognitive processing, such as face processing and attentional bias, as well as the comorbidity of SA and depression, through brain imaging technology, using brain signals as predictors of treatment outcomes in SA.

**Conclusion:**

This study conducted a comprehensive, objective, and visual analysis of publications, and revealed hot topics and trends concerning the study of the brain mechanism of SA from 1998 to 2023. This work might assist researchers in identifying new insights on potential collaborators and institutions, hot topics, and research directions.

## 1 Introduction

Social anxiety (SA) refers to the intense and persistent fear of social situations or engaging in behaviors that may be perceived as embarrassing (Hedman et al., 2013). It is estimated that about 10% of people may experience the disease in their lifetime (Alomari et al., 2022; Keller, 2003). Recent studies have shown that the global prevalence of SA is significantly higher than previously reported, with more than one-third (36%) of respondents meeting the threshold criteria for having SA (Jefferies and Ungar, 2020). The prevalence of SA symptoms among children, adolescents, and young adults (aged 6–25) in China is 23.5%, while in other countries, it ranges from 23 to 58% (Jefferies and Ungar, 2020). At present, SA has become one of the most common mental disorders.

SA co-occurs with many other disorders, such as depression disorder, anxiety disorder, and substance use disorder. The incidence of major depressive disorder (MDD) in SA patients is as high as 30–70% (Bas-Hoogendam et al., 2017). The comorbidity of the two disorders complicates treatment, leading to an increased risk of recurrence, substance use, and suicide (Dalrymple and Zimmerman, 2007; Liang et al., 2023). Therefore, accurately distinguishing the similarities and differences between different diseases has become a challenging issue and a development opportunity in the field of mental illness. With advancements in brain imaging technology, researchers have begun exploring the neural mechanisms underlying SA, which can not only provide objective neurobiological markers for clinical diagnosis but also explore the similarities and differences between different anxiety subtypes and other similar diseases (Kim and Yoon, 2018; Mizzi et al., 2022). This will provide more accurate therapeutic targets for future clinical diagnosis and treatment, greatly promoting the exploration of the pathogenesis, diagnosis, and treatment of SA.

At present, brain imaging studies on SA mainly focus on cognitive-related fields, such as emotional processing and attentional biases. Research on brain structure shows that SA is related to the volume of the amygdala, hippocampus, cingulate gyrus, and orbitofrontal cortex (Bas-Hoogendam et al., 2020; Brühl et al., 2014; Crane et al., 2021; Günther et al., 2020). Previous studies on brain activation have shown that the dysfunction of the amygdala, insula, hippocampus, and orbitofrontal lobe is related to SA. Studies on brain functional connectivity have found that patients with SA were characterized by the dysfunction of the frontal-limbic (fear) circuit, the hyperactivity of marginal areas (amygdala, hippocampus, and parahippocampal gyrus), and the low activity of cognitive control areas including the ventromedial prefrontal cortex, dorsolateral prefrontal cortex, and anterior cingulate cortex, which contributes to the inability to regulate SA effectively (Huang et al., 2022; Yu et al., 2021). Research has found a relationship between the characteristics of the brain and symptom networks in patients with SA, and the abnormality of the emotional network corresponds to the emotional disorder of patients. The abnormality of the default mode network indicates that patients have excessive self-reference thinking, the abnormality of the cognitive control network affects patients’ cognitive strategies, and the abnormality of the motivation network affects patients’ pleasant experiences in social activities (Kim and Yoon, 2018; Liu et al., 2024; Wang et al., 2020).

Most existing studies focus on a single research problem or subjectively summarize the main findings and progress within a specific field. At the same time, in the current research on the brain mechanism of SA, some results are contradictory due to some experimental errors (Yu et al., 2021). One of the more important reasons is that different investigators may choose different ROIs for the study, which may lead to inconsistencies and errors in the results. Due to the lack of a standardized methodology, there may be contradictions between the results of different studies, which makes the interpretation of these results difficult. Meta-analyses typically use the Activation Likelihood Estimation (ALE) method to explore SA’s brain mechanisms from a unified perspective. The results show that anxiety and depression have a common brain region, which will change after treatment (Liang et al., 2023). Moreover, SA, PTSD, and specific phobia all show the overactivation of the amygdala and insula in emotional-related research (Nicholson et al., 2016; Stefanescu et al., 2018; Stein et al., 2007). This shows that brain activation can be used to study the same and specific activities between different diseases. Most of the literature research methods adopt the traditional literature induction method and conduct research based on points of interest (ROI), which has a good effect in the in-depth literature mining, but there is also a certain subjectivity (Huang et al., 2022). This leads to the lack of systematic and intuitive presentation of the development context, research hotspots, and future research trends of the area, which cannot help readers understand the dynamics and development trends of this research field quickly and efficiently. Bibliometrics is a quantitative research discipline that employs mathematical and statistical methods to mitigate the subjectivity inherent in literature induction. Bibliometric analysis can visually analyze the history and present situation of a certain subject field with the help of statistical software such as CiteSpace, which transforms a large amount of complex and disorganized literature information into a structured and coherent knowledge system through analysis and summarization (Alajmi and Alhaji, 2018). In turn, it provides a relatively objective description and quantitative evaluation method for predicting the development trend of this field. It has become the primary tool for scholars to analyze the dynamics and development trends of research fields (Chen et al., 2024; Liu et al., 2024).

Therefore, this paper searched the Web of Science and Scopus databases and used CiteSpace software to visualize and analyze the relevant literature to reveal the hotspots in the field of brain mechanisms of SA and predict future research trends.

## 2 Methods

### 2.1 Data sources

In this study, we selected the Web of Science Core Collection and Scopus database to conduct a literature search on the brain mechanisms of SA from January 1998 to December 2023. The WoS and Scopus databases cover nearly the most comprehensive and authoritative scientific literature available globally and are widely used for bibliometric analysis and visualization of scientific literature (Sun et al., 2022).

The search terms of this paper refer to the published meta-analysis literature related to social anxiety and brain mechanisms (Cremers and Roelofs, 2016; Deng et al., 2023; Li et al., 2022; Sun et al., 2022). To make the studies more accurate and to further limit any bias that may have affected the results of the study, the research team conducted a comprehensive keyword search and manually screened and checked the preliminary results. The retrieval strategy was as follows: select advanced retrieval in the literature database of WoS and Scopus, using the following keywords: TS = (“social anxiety” or “social phobia” or “interaction anxiety” or “social avoid” or “social distress” or “social fear” or “fear of evaluation” or “fear of negative evaluation” or “fear of positive evaluation” or “communication anxiety” or “fear of rejection” or “social inhibition” or “social worry” or shyness or shy or “social avoidance distress” or “social anxiety” or “performance anxiety”) AND TS = (neuroimaging or “resting state” or “magnetic resonance elastography” or “MR elastography” or MRE or “structural magnetic resonance imaging” or sMRI or “functional magnetic resonance imaging” or fMRI or “magnetic resonance spectroscopy” or MRS or rs-fMRI or task fMRI or “near-infrared spectroscopy” or “NIR*” or “functional nearinfrared spectroscopy” or fNIRS or Electroencephalography or EEG or Electroencephalogram* or “quantitative electroencephalography” or QEEG or “quantitative EEG” or ERP or “event related potential” or “repetitive transcranial magnetic stimulation” or rTMS or “Transcranial Direct Current Stimulation” or transcranial direct current stimulation or TMS or tDCS or “positron emission tomography” or PET or “single-photon emission computed tomography” or SPECT or “functional connectivity” or “white matter” or “voxel based analysis” or VBM or “voxel based m orphometry” or “surface based m orphometry” or cortical or “diffusion-tens or imaging” or DTI). The TS tag performs a search of the given words in the title, abstract, keywords, and keywords Plus fields within a record.

**FIGURE 1:**
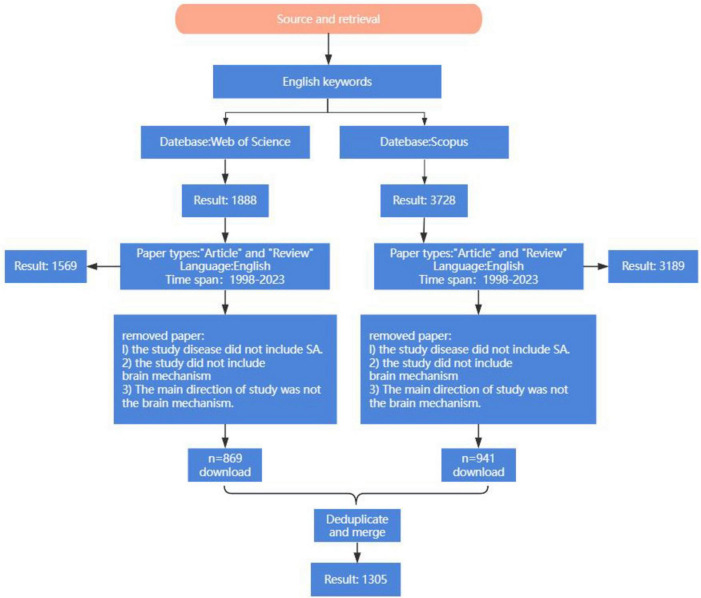
Flow chart of the research hotspots and trends of neuroimaging in SA.

There are a total of 5,527 articles. Literature types are limited to “Article” and “Review,” and the language of the document is selected as “English.” After the initial screening, 4,804 papers were obtained. Subsequently, two independent investigators reviewed the titles and abstracts, excluding articles not related to SA, which resulted in the inclusion of 1,305 publications. These retrieved studies were exported with complete records and cited references and imported into CiteSpace software. Additionally, the “Remove Duplicates” function was applied to reevaluate the data to ensure the validity of the information and accuracy of the results duplicate records were found. The formatted data files in the “output” folder were copied and pasted into the “data” folder for subsequent data analysis. The flow diagram of this study is shown in Figure 1.

### 2.2 Analysis tool

This study uses CiteSpace visualization software developed by Professor Chen Chaomei based on Java. CiteSpace can visually analyze the distribution, hotspots, and trends of a certain research field through the maps, thus effectively helping scholars master the recent research hotspots and frontier issues in this field. Nowadays, CiteSpace has been widely used in many research fields, such as psychology, medicine, and management (Zhang et al., 2022).

In this study, following the import of data into CiteSpace, the analysis period was established from January 1998 to December 2023. The number of published articles and collaborations was examined, and research hotspots related to the SA brain mechanisms were identified through keyword co-occurrence and clustering analyses. Additionally, keyword burst analysis and time zone diagrams were utilized to investigate future research trajectories and development directions.

In particular, the main outcome measures include keyword co-occurrence, keyword burst analysis, keyword cluster analysis, and keyword timezone analysis. When analyzing the map, keyword nodes represent the common keywords in literature research. At the same time, the node size represented the frequency of occurrence of the analysis object it represents. The more nodes, the richer the keywords in this field. Centrality is an index for measuring the importance of a node in a network, and high centrality is typically regarded as a pivotal point in a field. The purple circle represents centrality; the more comprehensive the circle is, the higher the centrality is. A node with high centrality is typically regarded as the pivotal point of a field (Liang et al., 2017). The larger the number of documents, the larger the node. The higher the centrality, the darker the color of the outer circle of the node. At the same time, the more connections between the node and other nodes, the closer the relationship between the two.

The centrality calculation uses the PageRank algorithm, which is based on the idea that the importance of a web page is determined by two factors: the number of other pages pointing to the page, and the quality of those pages. The method takes into account not only the number of surrounding points but also their quality.

The principle of co-occurrence analysis in CiteSpace is mainly based on the idea of “co-occurrence clustering.” It extracts information units in scientific literature, such as keywords, authors, institutions, etc., and reconstructs them according to the type and strength of the connection between these information units to form different network structures. In CiteSpace, co-occurrence analysis is one of its core functions.

The burst analysis method is based on Kleinberg (2002) proposal for a burst detection algorithm, the principle of which is to explore the research hotspots in the field by paying attention to the changes in the word frequency growth rate of a single word. It is generally believed that a word with a more incredible growth momentum indicates that it is more favored by researchers and is a more studied concern.

Cluster analysis is to cluster keywords by using spectral clustering algorithm. The algorithm is based on graph theory and is especially suitable for processing cluster problems based on link relation. By calculating the similarity and time proximity between keywords, keywords are clustered into multiple topic clusters.

The core of time zone map is to introduce time factor into the traditional co-occurrence network analysis, and to reveal the research hotspot and its evolution path through time division and combination analysis of co-occurrence network. Introduction of time factor: Based on the co-occurrence matrix, further increase the time information of the first occurrence of keywords. Specifically, the year in which each keyword first appeared in the dataset is recorded, and each keyword is labeled with a time label (Aydinoglu and Taskin, 2015; Cobo et al., 2011).

## 3 Results

### 3.1 Basic situation analysis

#### 3.1.1 Annual publication trends

**FIGURE 2:**
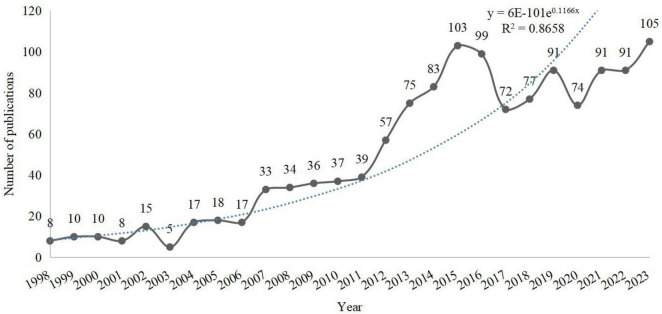
Annual number of publications.

Based on the fitted curve of the number of publications, it can be seen that the annual number of publications in the field of SA neuroimaging from 1998 to 2023 has generally shown an upward trajectory. Specifically, the number of annual publications before 2007 does not exceed 20. 2007–2011 maintains the number of annual publications at around 30. 2012–2015 was a period of rapid growth in the number of publications, with more than 100 in 2015. Although publication numbers fluctuated from 2016 to 2023, they remained consistently above 70 per year, reaching an all-time high of 105 publications in 2023. In summary, the field of SA neuroimaging has a promising research outlook with substantial potential for further growth (see Figure 2).

#### 3.1.2 Analysis by authors

After conducting a visualization analysis of the authors (Selection Criteria: g-index (*k* = 25), LRF = 3.0, L/N = 10, LBY = 5, *e* = 1.0, the co-occurrence plots below all use the same thresholds), there are 727 authors on the map. Table 1 shows the top ten authors with the highest publications and centrality. Of these, two have published more than 50 articles. The most prolific team is Pine, Daniel S, who has 59 publications related to the field. At the same time, this team has a centrality of 0.18, making it the most influential team. In addition, this result suggests that Fredrikson, Mats and Wei, Z should pay attention to the quality of their articles while increasing the number of publications. Aghajani, Moji has much potential in this area of research.

**TABLE 1:** The top 10 authors of neuroimaging in SA.

Rank	Author	Publication	Centrality	Year	Author	Publication	Centrality	Year
1	Pine, Daniel S	59	0.18	1999	Pine, Daniel S	59	0.18	1999
2	Phan, K Luan	58	0.05	2005	Aghajani, Moji	4	0.1	2012
3	Furmark, Tomas	38	0.08	1999	Gong, Qiyong	10	0.09	2010
4	Schmidt, Louis A	37	0.05	1999	Lui, Su	27	0.09	2015
5	Klumpp, Heide	37	0.06	2013	Furmark, Tomas	38	0.08	1999
6	Gong, Qiyong	27	0.09	2010	Straube, Thomas	24	0.07	2006
7	Fredrikson, Mats	27	0.00	2000	Leehr, Elisabeth J	6	0.07	2022
8	Fox, Nathan A.	26	0.05	1999	Klumpp, Heide	37	0.06	2013
9	Straube, Thomas	24	0.07	2006	Roelofs, Karin	7	0.06	2013
10	Zhang, Wei	22	0.01	2010	Phan, K Luan	58	0.05	2005

**FIGURE 3:**
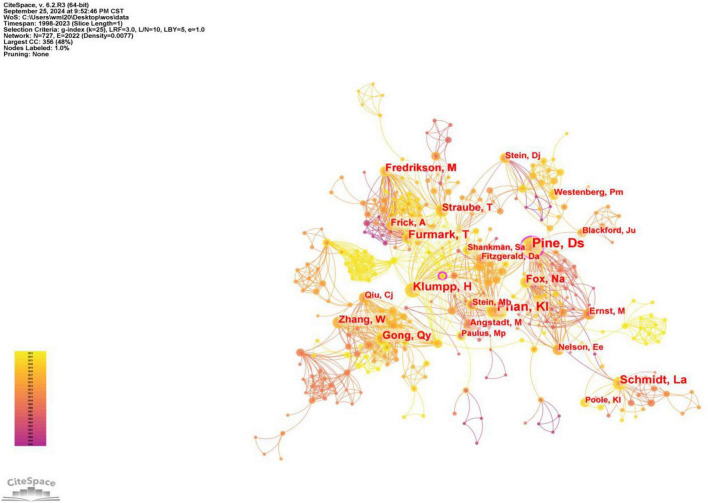
Map of authors of neuroimaging in SA.

As shown in Figure 3, the field mainly forms a cooperative group with high-productivity authors as the core and radiating outwards. Pine, DS occupies an central position and forms a network radiating outwards by influencing others through cooperation with others. Among the many collaborators of Pine, DS, there are relatively central authors, such as Phan, KI, Klumpp, H. Among them, Klumpp, H is associated with research teams led by Straube, T and Furmark, T. At the same time, Furmark, T is in contact with other researchers. Furmark, T collaborates with Lui, S, who is at the other center, and Lui, S collaborates with Zhang, W, who is at the center. In addition, some small and relatively independent research groups have appeared in the Atlas, such as those led by Gross, Jj, Miltner, Whr, and Westenberg, Pm.

#### 3.1.3 Analysis by institution

**FIGURE 4:**
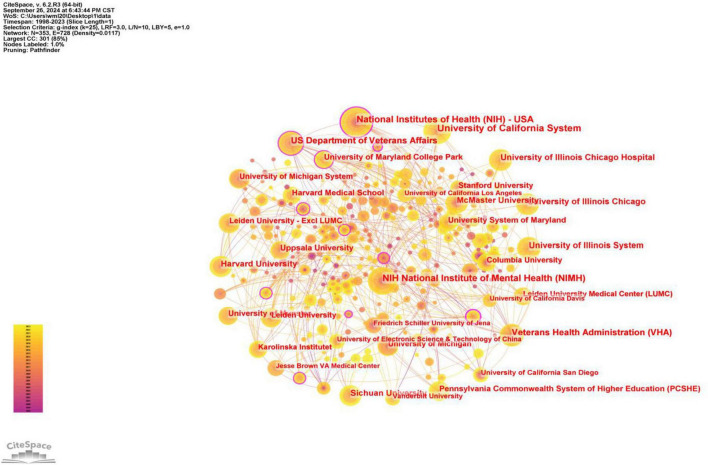
Map of the institution of neuroimaging in SA.

As can be seen in Figure 4, 319 institutions have participated in and published research on the neural mechanisms of SA, and a close collaborative network has been formed among the institutions, involving diverse institutions such as the National Institutes of Health, the US Department of Veterans, and the AffairsVeterans Health Administration, in addition to universities. This is conducive to improving the feasibility of research on brain mechanisms of SA and providing advanced equipment and resources. At the same time, cross-institutional collaboration can bring different perspectives and innovative ideas to research, avoid limitations in thinking, promote multidisciplinary synergistic research, and facilitate the generation of breakthrough results. Specifically, three institutions published more than 50 papers, and the rest had more than 30 publications. Among them, Harvard University has the highest centrality, having published relevant papers since 2001, with a centrality of 0.17. Many institutions and groups are highly focused on the advancements in the neural mechanisms of SA and continue to conduct research. These institutions have conducted a large number of studies on brain mechanisms in the early stage of this field, which has certain forward-looking and reference significance. The information related to the top ten institutions is shown in Table 2 below.

**TABLE 2:** The top 10 institutions of neuroimaging in SA.

Rank	Institution	Count	Time of first posting	Institution	Centrality	Time of first posting
1	University of California System	70	1998	Harvard University	0.17	2001
2	National Institutes of Health	60	1999	Humboldt University of Berlin	0.15	1998
3	NIH National Institute of Mental Health	56	1999	National Institutes of Health (NIH) - USA	0.13	1999
4	US Department of Veterans Affairs	47	2002	University of California System	0.1	1998
5	Veterans Health Administration	46	2004	NIH National Institute of Mental Health (NIMH)	0.09	1999
6	University of Illinois System	42	2005	Karolinska Institutet	0.09	2007
7	Harvard University	42	2001	Beijing Normal University	0.09	2014
8	University of Illinois Chicago	39	2013	US Department of Veterans Affairs	0.07	2002
9	University of Illinois Chicago Hospital	39	2013	Veterans Health Administration (VHA)	0.07	2004
10	McMaster University	36	1999	Columbia University	0.07	1998

#### 3.1.4 Analysis of journal co-citation

Co-cited journals were journals cited together by researchers, which usually reflected the foundation of a research field and were one of the most critical indicators in bibliometric analysis. As can be seen in Table 3, the top three co-cited journals are Biological Psychiatry, with an IF of 9.6 and cited 661 times; Neuroimage, with an IF of 4.7 and cited 653 times; American Journal of Psychiatry, with an IF of 15.1 and cited 588 times. The top three in centrality are Biological Psychology, with an IF of 2.7 and a centrality of 0. 06; Annals of The New York Academy of Science, with an IF of 4.1 and a centrality of 0.05; Child Development, with an IF of 3.9 and a centrality of 0.05, which suggests these journals were well the most influential in this field.

**TABLE 3:** The top 10 co-cited journals of neuroimaging in SA.

Rank	Frequency	Centrality	Co-cited Journal	If (2023)	Frequency	Centrality	Co-cited Journal	If (2023)
1	661	0.00	Biological Psychiatry	9.6	376	0.06	Biological Psychology	2.7
2	653	0.01	Neuroimage	4.7	217	0.05	Annals of The New York Academy of Sciences	4.1
3	588	0.01	American Journal of Psychiatry	15.1	126	0.05	Child Development	3.9
4	576	0.01	Archives of General Psychiatry	/	72	0.05	American Psychologist	12.3
5	466	0.01	Journal of Neuroscience	4.4	70	0.05	European Archives of Psychiatry and Clinical Neuroscience	3.5
6	435	0.03	Behavior Research And Therapy	4.2	412	0.04	Psychological Medicine	5.9
7	412	0.04	Psychological Medicine	5.9	136	0.04	Current Opinion in Neurobiology	4.8
8	397	0.02	Depression And Anxiety	4.7	62	0.04	Journal of Neuropsychiatry and Clinical Neurosciences	2.4
9	392	0.00	Human Brain Mapping	3.5	435	0.03	Behavior Research and Therapy	4.2
10	383	0.01	Trends in Cognitive Sciences	16.7	347	0.03	Psychiatry Research-Neuroimaging	2.1

#### 3.1.5 Analysis of references co-citation

Table 4 demonstrates the ten articles with the highest total number of citations in the field of SA neuroimaging. These frequently cited references are considered landmark literature in the field and significantly support subsequent research. Overall, the topics of these highly cited references mainly focus on “meta-analysis,” “amygdala,” “emotional processing,” and “cognitive regulation.” This suggests that future scholars should pay more attention to these research topics in the field.

**TABLE 4:** The top 10 references of neuroimaging in SA.

Rank	Cited frequency	Year	Title	Journal	IF(2023)
1	64	2014	Neuroimaging in social anxiety disorder-a meta-analytic review resulting in a new neurofunctional model	Neuroscience and Biobehavioral Reviews	7.5
2	54	2006	Association between amygdala hyperactivity to harsh faces and severity of social anxiety in generalized social phobia	Biological Psychiatry	9.6
3	52	2010	Neuroimaging in social anxiety disorder: a systematic review of the literature	Progress in Neuro-psychopharmacology & Biological Psychiatry	5.3
4	52	2007	Functional neuroimaging of anxiety: a meta-analysis of emotional processing in PTSD, social anxiety disorder, and specific phobia	American Journal of Psychiatry	15.1
5	51	2009	Neural bases of social anxiety disorder: emotional reactivity and cognitive regulation during social and physical threat	Archives of General Psychiatry	/
6	44	2011	Reduced resting-state functional connectivity between amygdala and orbitofrontal cortex in social anxiety disorder	NeuroImage	4.7
7	36	2011	Emotional processing in anterior cingulate and medial prefrontal cortex	Trends in Cognitive Sciences	16.7
8	35	2011	Diagnostic and Statistical Manual of Mental Disorders	Psychiatry Research	4.2
9	33	2013	Aberrant amygdala-frontal cortex connectivity during perception of fearful faces and at rest in generalized social anxiety disorder	Depression and Anxiety	4.7
10	30	2011	Neural correlates of altered general emotion processing in social anxiety disorder	Brain Research	2.7

### 3.2 Research hotspot analysis

Research hotspots refer to topics frequently emphasized in interrelated clusters of literature, which are intrinsically connected and appear within a specific time frame (Zhang et al., 2022). Analyzing research hotspots helps us understand the topics scholars focus on in a specific period and clarifies the development context of these topics. Keywords, as condensed summaries of literature content, serve to interpret and express the main themes of research. In visual research studies, topic keyword co-occurrence and cluster analysis are commonly used to identify research hotspots.

Based on co-word analysis, extracting the distribution of keyword frequencies from literature information can identify research hotspots in a specific field (Xu et al., 2024). High-frequency keywords reflect the core concern in this research field, which is related to advanced research methods and problems to be solved in a certain period (Xu et al., 2024). Co-word clustering analysis is used to identify the scientific clusters of high-frequency keywords and analyze the co-occurrence matrix of keywords, which can determine the evolution of the relationship in the research literature. Therefore, analyzing the keywords of a field can provide insights into its research hotspots, which is immensely significant for researchers and constitutes an important aspect of visual analytics.

#### 3.2.1 Keyword co-occurrence analysis

**FIGURE 5:**
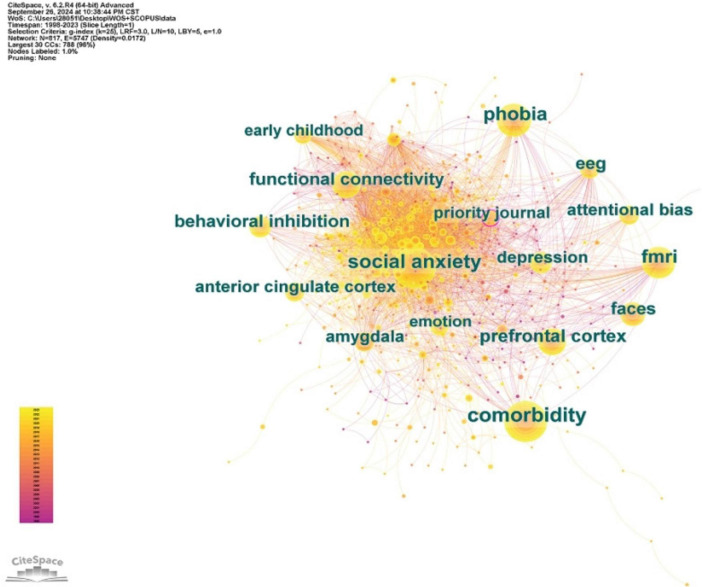
Map of keywords in research hotspots and trends of neuroimaging in SA.

Through CiteSpace, we conducted a visual analysis of keywords. We selected the top ten keywords ranked by frequency and centrality (Table 4). For better understanding, these keywords can be roughly divided into four categories: research techniques (functional connectivity, fMRI, EEG, PET), comorbidities (comorbidity, depression), areas of interest in the brain (amygdala, prefrontal cortex, anterior cingulate cortex, brain mapping), and research fields (faces, behavioral inhibition, attentional bias). The results in Table 5 are further validated by the co-occurrence map shown in Figure 5.

**TABLE 5:** The top 10 keywords in occurrences frequency and centrality for research hotspots and trends of neuroimaging in SA.

Rank	Keywords	Frequency	Keywords	Centrality
1	Comorbidity	547	Amygdala	0.07
2	fMRI	365	Anterior cingulate cortex	0.06
3	Prefrontal cortex	291	Early childhood	0.06
4	Functional connectivity	235	Activation	0.06
5	Faces	222	Brain mapping	0.06
6	EEG	210	Brain cortex	0. 6
7	Behavioral inhibition	208	fMRI	0.05
8	Depression	192	EEG	0.05
9	Amygdala	178	Behavioral inhibition	0.05
10	Attentional bias	171	PET	0.05

From these keywords, we can ascertain that fMRI is the primary technology currently relied upon in researching the neural mechanisms of SA. A multitude of functional magnetic resonance imaging (fMRI) studies have provided crucial information on the neurophysiological mechanisms of SA at the brain function level (Picó-Pérez et al., 2023; Pierce and Black, 2023; Zugman et al., 2023). Studies have suggested that a characteristic of SA patients is the dysfunction of the limbic-prefrontal circuit, particularly centering around the prefrontal cortex, and amygdala (Jafari et al., 2021; Mao et al., 2020). In recent years, with the advancement of research techniques, an increasing number of studies have employed resting-state and task-based functional magnetic resonance imaging methods to investigate the abnormalities in functional connectivity patterns among individuals with SA (He et al., 2024; Pierce and Black, 2023). Research has found that abnormal insula-prefrontal connectivity indicates that individuals with SA exhibit a hyperresponsive reaction to threatening stimuli compared to healthy controls (Klumpp et al., 2012). These studies contribute to understanding the neural mechanisms of SA.

In addition, exploring the comorbidity of SA and depression using brain imaging technology is also a hot research topic (He et al., 2024). This is because comorbidities are prevalent in people with SA, occurring in up to 90% of cases. Comorbidities pose significant etiologic and diagnostic difficulties and can create treatment challenges. Increasing attention is being paid to the unique and shared neuroanatomical features of Major depressive disorder(MDD) and SA (Fayazi and Hasani, 2017; Steiger et al., 2017). A meta-analysis suggested that reduced GM volume in the right parahippocampal gyrus, particularly in the amygdala, may be associated with a high rate of co-morbidity between MDD and SA as well as similar cognitive patterns (Pei et al., 2023). In addition, the current meta-analysis identified a distinct pattern of GM reduction, with lower GM volumes in the right pupillary nucleus and medial frontal gyrus in patients with SA and the left parahippocampal gyrus in patients with MDD. This pattern of GM volume reduction is consistent with the clinical presentation of MDD and SA. These findings enhance our understanding of the underlying neuropathogenesis of MDD and SA, offering potential imaging markers for both disorders (Liang et al., 2023).

Some researchers have used fMRI techniques to explore the activity characteristics of the corresponding brain regions for cognitive processing in socially anxious individuals (Evans et al., 2020; Koban et al., 2023; Lin et al., 2023). For example, some researchers have used the fMRI-based point-probe paradigm to reveal distinct patterns of threat-related attention in individuals with SA. It was found that the process of avoidant attention and slow disengagement from the threat was associated with the deactivation of the default mode network and a stronger connection of the amygdala to the superior temporal sulcus compared to vigilant orientation and rapid disengagement. This suggests that different neural processes contribute to distinct patterns of threat-related attention in SA (Evans et al., 2020). It was also found that a group of patients with SA showed significant attention retention effects only to angry face distractors. Neural activity in the amygdala, insula/inferior frontal gyrus (IFG), and temporoparietal junction (TPJ) was increased in patients with SA compared to controls with sudden onset of angry distractors. Brain regions associated with attentional redirection to distractors (TPJ and IFG) also maintained higher activity in SA patients, which is consistent with their behavioral findings regarding the attention retention effect (Kim et al., 2018). This suggests that patients with SA exhibit a long-term attentional bias toward task-unrelated social threats, with the underlying mechanism manifested as overactivity of the amygdala and persistent activity of bottom-up attention networks, including TPJ and IFG (Kim et al., 2018; Santos et al., 2019).

From the perspective of research fields, face processing, behavioral inhibition, and attentional bias are currently hot research topics. In addition to high-spatial-resolution fMRI brain imaging technology, researchers prefer to use high-temporal-resolution ERP to investigate the neural physiological mechanism of SA face processing (Tian et al., 2024). Utilizing high-temporal resolution ERP technology to investigate the processing of dynamic emotional faces with high ecological validity in individuals with SA can help elucidate the essence of emotional processing in this population, providing more substantial evidence for models of facial emotion processing (Herrmann et al., 2005). The model of facial emotion processing suggests that face processing includes four stages, each involving different ERP components. Specifically: Emotional faces capture attention (P1, attention capture process) → Face structure encoding (N170, face perception process) → Integration of low-level and structural information (ERN, selective attention process to facial emotions based on certain features) → Ongoing deep processing of specific emotions (LPP, evaluation process) (Schindler and Bublatzky, 2020). Existing studies have found that the ERP components related to the processing of emotional faces in individuals with high SA include P100, P200, N170, P300, ENP, LPP, LPC, etc (Günther et al., 2021; Pei et al., 2023; Schindler and Bublatzky, 2020). The P100 component of people with high SA was enhanced, indicating that they were more alert to emotional stimuli in the early stage of expression processing. The P200 wave is only stronger when processing angry expressions, which means that it has an anger evaluation bias, and more cognitive resources need to be consumed when evaluating angry expressions. The P300, LPP, and LPC components were enhanced when people with high SA processed expressions such as threat and anger, indicating that they processed all negative expressions more deeply and had more difficulty detaching from them (Botelho et al., 2023; Pei et al., 2023; Tian et al., 2024). These studies show that SA patients have a significant selective attention bias toward threats, which is manifested in the fact that they are more likely to notice threatening information and more difficult to divert attention from threatening information. The aberrant attentional processing theory proposes that there is an initial over-vigilance or facilitation of the hypothesis of clinically relevant threats and subsequent defensive avoidance in SA and, more broadly, anxiety disorders. Some studies have used steady-state visual evoked potential (ssVEP) as an indicator of attention allocation to investigate the time course of changes in socially anxious subjects’ attention bias to threatening stimuli (McTeague et al., 2018). The results showed that socially anxious patients exhibited the most significant ssVEP amplitude in response to aversive facial expressions and sustained visual cortical ease, suggesting that socially anxious patients exhibited a persistent pattern of hypervigilance. Other researchers further explored the dynamic attention patterns of socially anxious individuals toward threatening social stimuli, validating and expanding the discussion on the relationship between anxiety, attentional control abilities, and negative attention bias within the framework of attentional control theory. The results showed that the high SA was more sensitive than the happy and neutral faces. The amplitude of ssVEP induced by consistently angry faces was the highest (Zheng et al., 2023). At the same time, the scores of attention concentration and attention transfer ability were negatively correlated with the scores of SA. There was a significant negative correlation between attention transfer ability score and negative attention bias index. This study revealed the attention pattern of socially anxious individuals to threatening stimuli; that is, they initially showed attention vigilance to threatening social stimuli, then failed to make adaptive avoidance due to impaired attention control ability, and finally showed difficulty in attention detachment (Zheng et al., 2023). Negative bias toward threatening stimuli and poor control may be risk factors for the emergence and development of SA symptoms (Botelho et al., 2023). This will help to understand further the pathogenesis of the emergence and maintenance of SA and develop more targeted and effective forms of treatment, providing a theoretical basis and empirical support for intervention strategies of SA.

Moreover, SA is the developmental outcome most frequently studied in behaviorally inhibited children, with prolonged high levels of behavioral inhibition leading to a vulnerability to SA (Fox et al., 2021; Thai et al., 2016). Additionally, research has found that behaviorally inhibited children are at greater risk for developing psychological disorders in the future due to similar neural processes or structural characteristics to certain individuals with SA. The identification of these neural process associations has laid the foundation for cognitive neuroprocesses as predictors of risk in behaviorally inhibited children. Roy et al. (2014) focused on the extensive amygdala system. This article suggests that functional disorders in the amygdala, prefrontal cortex, striatum, anterior insula, and cerebellum are risk indicators for anxiety disorders in behaviorally inhibited individuals. Previous ERP studies on attention processes in behaviorally inhibited individuals have also focused on the P1, N2, et al. Components (Thai et al., 2016). For instance, Jetha et al. (2012) found in adult subjects that shy individuals exhibited a reduction in the P1 component when confronted with emotional faces (Jetha et al., 2012). Researchers believe this is due to the cognitive avoidance and attentional inhibition of socially anxious individuals. This study confirmed the association between shyness and early cortical responses to fearful faces, consistent with the amygdala sensitivity model. The study also found that early behavioral inhibition in children can predict an increase in socially specific ERN and the emergence of SA symptoms in adolescence. The increase in ERN may be due to the overactive fear system in behaviorally inhibited children, requiring higher levels of inhibitory control (Lahat et al., 2014; McDermott et al., 2009). The ERN may be a neurobehavioral mechanism that links behavioral inhibition to adolescent SA symptoms and diagnosis. Further research has found that the connection between behavioral inhibition and SA is influenced by attentional control and attentional bias (Gilbert et al., 2022). Studies have assessed whether ERP responses related to attention to angry and happy faces mediate the longitudinal association between BI and SA in preschool children. The results showed that P1 responses to happy faces and N2 responses to angry faces mediated the relationship between behavioral inhibition and SA. P2 and N2 amplitudes were associated with SA and attentional bias, respectively. This highlights the importance of observing individual differences in the developmental study of neural predictors of SA (Thai et al., 2016). Future research could further explore effective intervention methods based on the cognitive and neural processes of behaviorally inhibited individuals, helping children reduce the risk of social withdrawal and psychological disorders.

#### 3.2.2 Keyword cluster analysis

The article selected a g-index of 25 for clustering analysis. The keywords in the literature are highly concise, summarizing the content and theme of the literature. Therefore, through the keyword clustering research analysis, we can better understand the relevant direction and hot spots in this field (Xu et al., 2024).

**FIGURE 6:**
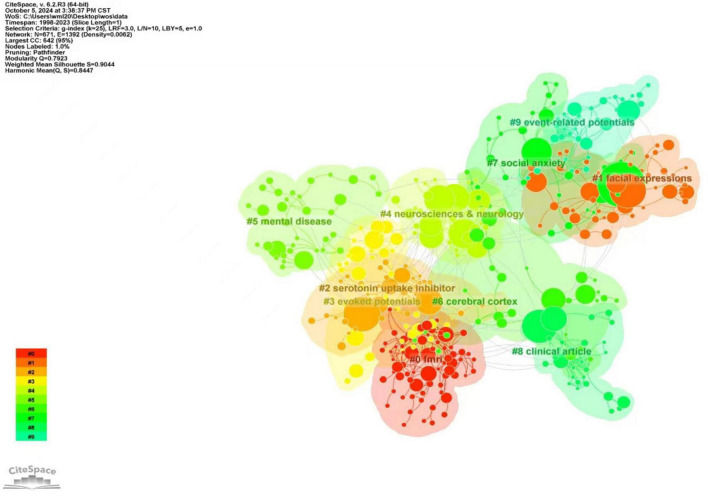
Clustering map of keywords about research hotspots and trends of neuroimaging in SA.

The map displays the top ten cluster labels. Figure 6 indicates that the subject terms related to retrieval strategies were removed. A more detailed clustering is shown in Table 6, where the research can be divided into four main areas. Among them, #0, #3, and #9 can be classified as research techniques for the neurophysiological mechanisms of SA; #1 can be categorized as the neurophysiological mechanisms of cognitive processing in SA; #6 and #7 can be grouped as the core brain regions, and networks focused on in SA brain imaging research. #2, #4, #5, and #8 can be classified as the neural predictors of treatment outcomes in SA. The following analysis will explore the current hotspots in research from three aspects: research techniques for the neurophysiological mechanisms of SA, research fields, core brain regions and their networks, and the neural predictors of treatment outcomes in SA.

**TABLE 6:** Cluster analysis of SA brain mechanism studies.

Cluster ID	Size	Silhouette	Mean (year)	Label (LLR)
0	69	0.89	2009	**fMRI;** bipolar disorder; mood disorders; functional neuroimaging; tic;
1	50	0.829	2007	**Facial expressions;** amygdala activation; functional MRI; medial prefrontal cortex; cerebral blood-flow;
2	43	0.94	2007	**Serotonin uptake inhibitor;** 4 aminobutyric acid; treatment response; monoamine oxidase inhibitor; benzodiazepine derivative;
3	41	0.851	2016	**Evoked potentials;** evoked response; event-related potential; neurosciences & neurology; electroencephalography;
4	39	0.895	2008	**Neurosciences & neurology;** psychiatry; obsessive-compulsive disorder; posttraumatic stress disorder; nuclear magnetic resonance imaging
5	35	0.935	2017	**Mental disease;** transcranial direct current stimulation; quality of life; cognitive behavioral therapy; test-retest reliability;
6	35	0.857	2013	**Cerebral cortex;** brain cortex; default mode network; generalized social phobia; neural bases;
7	35	0.93	2013	**Social anxiety;** anterior cingulate cortex; generalized anxiety; functional connectivity; psychiatry;
8	33	0.949	2006	**Clinical article;** neurosciences & neurology; psychiatry; controlled study; double-blind procedure;
9	32	0.956	2009	**Event-related potentials;** trait anxiety; selective attention; psychology; ERP;

(1) Research methods mainly include fMRI, EEG, and Event-related potentials. This shows that fMRI, EEG, and Event-related potentials (ERP) are commonly used techniques in SA research (Al-Ezzi et al., 2020; Mizzi et al., 2022; Parsaei et al., 2024; Pei et al., 2023). Among them, fMRI can reveal the brain mechanisms underlying social disorders with high spatial resolution, accurately differentiating similar conditions at the neurobiological level and thereby assisting in clinical diagnosis and treatment (Peterson et al., 2014). Especially in clinical practice, the identification of comorbidities is very vital. The presence of one disease may mask another or lead to more complex clinical manifestations, increasing the risk of misdiagnosis and possibly leading to poorer treatment outcomes (Koyuncu et al., 2019). About half of people with SA have comorbidities of mental illness, drug addiction, or alcohol problems (Robichaud and Koerner, 2019). SA has long been shown to suffer from comorbidities, with post-traumatic stress disorder (PTSD), obsessive-compulsive disorder (OCD), and SA all carrying core symptoms of anxiety (Cheng et al., 2015). Comorbidities not only hinder treatment but also increase the risk of suicide in patients with these diseases, so precise differentiation is essential for clinical treatment (Rozen et al., 2022). Researchers use fMRI to study mood disorders, such as depression and bipolar disorder, and distinguish the difference between SA and other diseases, which is conducive to the diagnosis and treatment of SA (He et al., 2024; Liang et al., 2023). For example, one fMRI study compared gray matter volume (GMV) in magnetic resonance images obtained from PTSD patients, OCD patients, SA patients, and healthy controls. GMV was different in the left hypothalamus and left inferior parietal lobe in all four groups, and this difference was mainly due to reduced GMV in the PTSD group relative to the other groups. The PTSD group exhibited decreased GMV in the frontal, temporal, and cerebellar lobes compared to the OCD group, as well as reduced GMV in the bilateral frontal lobes compared to the SA group. Additionally, GMV in the left hypothalamus showed a significant negative association with anxiety symptoms in all three disorder groups (Cheng et al., 2015). The study also found evidence of structural differences in the brain that provide biomarkers for the diagnosis of SA and support the idea of using MRI in the clinical classification of these diseases (Al-Ezzi et al., 2020).

In addition, EEG is also an effective way to study neural correlates of SA and to obtain large-scale connectivity models of brain function. Among them, the spectral coupling between delta and beta oscillations is associated with SA (Al-Ezzi et al., 2020). At the same time, EEG bands were associated with different functional and behavioral correlations. For example, slow wave (SW) brain oscillations such as delta are associated with subcortical regions responsible for motivation, emotion, and reward processing (Knyazev, 2012; Newson and Thiagarajan, 2019). In contrast, fast waves reflect intercortical connections that are activated when attention is needed for control, cognitive processing, and regulation (Poole et al., 2021). It has been found that longitudinal patterns of neuroendocrine stress activity and SA in early childhood may be related to the EEG power of slow and fast brain oscillations in the frontal lobe (Poole and Schmidt, 2019). Meanwhile, previous studies have found evidence that the level of spectral coupling between frontal SW and FW (fast wave) can be used to distinguish socially anxious individuals and quantify the severity of SA during social interactions (Miskovic and Schmidt, 2010).

The evoked potential is the third major development in clinical neurophysiology after electroencephalogram and electromyography, and it can help diagnose the abnormal function of the nervous system (Knyazev, 2012). The event-related potential is an exceptional evoked potential which can objectively reflect the cognitive processing of the brain. Event-related potentials (ERPs) are direct measures of brain activity that can be correlated with individual differences, helping to clarify the similarities and differences between seemingly related disorders and their features (Hajcak et al., 2019). ERP research on SA mainly involves attention bias, cognitive control, emotional response, and other cognitive processing processes involving P1, N170, N2, ERN, FPN, LPP, and other EEG components (Al-Ezzi et al., 2020; Pei et al., 2023). Among them, P1 and N170 are mainly related to bottom-up automatic processing of information and are often used to explore attention bias, early emotional information, and face processing in patients with SA and anxiety disorders (Song et al., 2022). ERN, FRN, and N2 involve the top-down monitoring process of internal and external threat information (Pei et al., 2023). One ERP study showed that healthy people showed more significant adjustment of late components, such as changes in P3 components, compared to anxious people. Index conscious and assessed processing of threats and emotions and post-processing disengagement difficulties (Gupta et al., 2019). This phenomenon is indicative of SA patients’ awareness and evaluation of threat and emotion processing and disengagement difficulties in the later stages of processing. The ERP component was also able to predict changes in SA symptoms. For example, multiple studies have demonstrated the unique predictive power of ERN in describing how children will become anxious at ages 6–9, i.e., ERP can be used to predict changes in diagnostic status (Filippi et al., 2020). Other studies have suggested that ERPs may also help predict changes in symptom dimensions. At the same time, ERPs can predict treatment outcomes. For example, attention deviation correction (ABM) is a potential intervention for alleviating symptoms of SA. An ERP study intervened for attention bias in SA by modulating the early allocation of attention to material-related stimuli. The results showed that the training group showed similar reductions in late positive latency (LPP), and the reduction of LPP was positively associated with improvements in behavior and symptoms (Pan et al., 2019). Other studies have found that the attention bias correction task can induce the vMMN component, and this component can effectively predict the clinical treatment effect of SA (Arad et al., 2019).

Neuroimaging techniques, such as fMRI, EEG, and event-related potentials (ERP), have critical applications in the clinical diagnosis and treatment of SA (Al-Ezzi et al., 2020). By providing high spatial resolution images of the brain, fMRI can reveal differences in brain function in patients with SA and help distinguish SA from other comorbidities (such as PTSD and OCD), thereby providing accurate diagnostic markers for clinical use, avoiding misdiagnosis and optimizing treatment (Pannekoek et al., 2015). EEG helps assess the functional status of SA patients by monitoring the coupling relationship between brain waves (such as delta waves and beta waves) and provides a basis for personalized treatment. For example, EEG can identify patterns of a patient’s neural response in the face of a social threat, which can guide the clinical selection of appropriate treatment (Al-Ezzi et al., 2020). ERP analyzes EEG components such as P1, N170, and ERN to reveal the differences in cognitive processing in social situations in SA patients, which is helpful for early diagnosis and prediction of treatment response. For example, ERP-based attention bias training is effective in reducing SA symptoms and predicting treatment outcomes (Hajcak et al., 2019). In conclusion, neuroimaging technology not only promotes the accuracy of SA diagnosis but also provides new tools for clinical treatment, especially in the identification of comorbidities and personalized treatment, which improves the scientificity and effectiveness of clinical decision-making.

(2) Research fields mainly include face processing. Emotional faces are among the most common emotional stimuli in daily life, conveying information related to physical and psychological states, such as age, emotion, purpose, and desire. They hold significant social and evolutionary importance (Lacombe et al., 2023). Heightened sensitivity to facial expressions is often observed in SA, especially those that are threatening or scrutinizing. A classic symptom of SA is an increased emotional reactivity to potential social threats, showing a persistent pattern of hypervigilance (Chen et al., 2020; Wauthia et al., 2023). The abnormal processing of facial expressions may be a significant cause of social communication disorders. Using brain imaging techniques, the researchers found that SA was associated with abnormal amygdala responses to threat-related stimuli (Demenescu et al., 2013). One study examined and confirmed the hypothesis that the high reactivity of the amygdala check-in faces conditioned on social evaluative significance is a candidate for the endophoric phenotype of SA (Bas-Hoogendam et al., 2020). The prefrontal area is involved in the attention and processing of facial features (Clark et al., 2015). A meta-analysis showed that the face elicited a higher SA response in the bilateral amygdala, pallidum, superior temporal sulcus, visual cortex, and prefrontal cortex (Gentili et al., 2016). For example, a study using emotional faces as stimulus information found that socially anxious individuals showed higher activation of the left and right amygdala than control samples when stimulated with high-stress faces (Yoon et al., 2007). Crane et al. (2021) explored neural differences in socially anxious individuals when confronted with different emotional faces using emotional faces as stimulus material. Results showed that anxious individuals had higher amygdala activation when dealing with angry faces (Crane et al., 2021). Günther et al. (2020) measured the automatic responses of the amygdala in anxious individuals when confronted with emotional faces using fMRI, confirming the central role of the amygdala’s response to unconsciously perceived threats in understanding and predicting personality anxiety. Meanwhile, a study verified the high responsiveness of the amygdala to faces conditioned with socially evaluative meaning, validating that the amygdala is involved in responding to conditioned faces with socially evaluative meaning and is an important research brain region and clinical indicator of SA (Bas-Hoogendam et al., 2020). Meanwhile, the connection between the amygdala and the medial prefrontal cortex (mPFC) was found to be associated with anxiety (Liu et al., 2020). The amygdaloid-MPFC connection plays a vital role in the diagnosis of SA. When anxiety is negatively associated with the amygdaloid-ventral mPFC connection, it indicates impaired emotional regulation in the individual, while a positive association with the amygdaloid-dorsal mPFC connection indicates heightened alertness to external stimuli (Porta-Casteràs et al., 2020; Sun et al., 2023). In a study using fMRI to explore performance differences between individuals with SA and healthy controls on facial emotion-processing tasks, researchers found that patients with SA exhibited overactivation of the amygdala, along with reduced functional coupling of the left amygdala with the medial orbitofrontal cortex and the posterior cingulate cortex/precuneus during emotional tasks The strength of this functional connection was inversely correlated with the severity of state anxiety (Hahn et al., 2011).

The current research is to examine the neural mechanisms of SA to find out how to objectively diagnose patients and provide individualized treatment recommendations. Reliable and effective biomarkers provide a solid basis for the diagnosis and treatment of SA (Al-Ezzi et al., 2020). To prevent and treat SA more effectively, researchers have been exploring the pathophysiological mechanism of SA. Early explorations focused on individual brain regions related to self and emotion, such as the prefrontal cortex, amygdala, and cingulate gyrus (Freitas-Ferrari et al., 2010). With the continued advancement of research, particularly in fMRI and other technologies, some scholars have suggested exploring changes in connectivity patterns between different brain regions from the perspective of brain networks. We can better understand the role of specific brain regions in the formation and development of mental disorders such as SA, and also better understand the neurobiological mechanisms behind it, rather than just looking at reduced activity in a single brain region (Mizzi et al., 2022, 2024).

Studies have shown that abnormal processing of facial expressions in SA has important clinical significance, especially sensitivity to threatening or scrutinizing expressions (Chen et al., 2020). Through neuroimaging techniques, studies have found that SA patients have abnormal activation of brain regions such as the amygdala when faced with emotional faces, especially when stimulated by threatening faces, which provides the potential for biomarkers in clinical practice (Demenescu et al., 2013). At the same time, the connection pattern between amygdala and prefrontal cortex is closely related to the ability to regulate emotions, which provides a new idea for personalized treatment (Bas-Hoogendam et al., 2020). In recent years, neural network research has also highlighted changes in connectivity between brain regions, helping to understand the formation and development of SA more comprehensively (Sun et al., 2023). Therefore, the study based on brain activity and connectivity patterns can promote the early diagnosis of SA and the formulation of personalized treatment plans, improve the treatment effect, and further open up a new direction of neuroscience and clinical treatment.

(3)The core brain regions and networks mainly include the default network (DMN) and the anterior cingulate cortex (ACC). So far, researchers studying the brain mechanisms of SA have identified several brain networks associated with SA, including the central executive network, default network, and dorsal attention network, with the prefrontal cortex and amygdala as core regions. Additionally, they have found functional connectivity abnormalities between the amygdala and the prefrontal cortex, which may serve as important neural representations of cognitive regulation failure, biases in self-information processing, and attention biases in SA (Caldiroli et al., 2023; Klumpp et al., 2014). These findings show promise as potential biomarkers.

Among them, DMN is one of the classical resting state brain networks and the most studied network (Kim and Yoon, 2018; Wang et al., 2020). The DMN is a set of time-related brain regions that are most active at rest and deactivated when performing cognitively demanding goal-oriented tasks. This network includes the medial prefrontal cortex (mPFC), the posterior cingulate cortex (PCC)/anterior cuneus, and the ventral/pregenicular cingulate cortex (pgACC). Default mode network plays an important role in individuals’ social cognition and self-referential processing (SRP), and is also closely related to social-emotional processing (Jamieson et al., 2023; Yoon et al., 2019). It is believed that the functional connectivity of the DMN region is abnormal in SA patients due to high self-attention. The functional connectivity of DMN in mild to moderate SA patients is enhanced, while the functional connectivity of DMN in severe SA patients is decreased (Liao et al., 2010; Zhang et al., 2023). It has also been shown that low RS-connectivity between DMN regions is inversely associated with task-non-specific hyperactivity in DMN and amygdala (AMG)/SN. At the same time, we propose a model of “anxious self-terrain” in SA (TAS-SA). The model shows that the abnormal DMN-AMG/SN topography observed in individuals with SA at rest, which reflects an “unstable social self,” is significantly aggravated in sensitive patients, leading to increased activity in the relevant brain networks (Lucherini Angeletti et al., 2023). The high connectivity of the anterior cingulate cortex (ACC) and the medial prefrontal cortex (mPFC), the core brain regions of the default network, to the amygdala also suggests the relevance of this region to threat processing in SA (Lucherini Angeletti et al., 2023). At the same time, an enhanced functional connection between the bilateral dorsal ACC and PCUN was also found in SA patients. This enhanced connection links CN with DMN and may be the neural basis for the enhanced attention of SA patients to self-related information in the external environment (Pannekoek et al., 2013). Liao et al. (2010) found that the functional connectivity of the dorsal anterior cingulate cortex (ACC) and middle cingulate cortex (MCC) is enhanced in patients with SA. This connectivity is significantly positively correlated with scores on the Leibowitz SA Scale, further suggesting that there may be an enhanced effect of brain region connectivity associated with self-related representation in SA (Liao et al., 2010). Therefore, the enhancement of connections in the cingulate subregion may be one of the crucial signs that SA has a self-focused attention bias to external information.

In addition, people with anxiety disorders are likely to experience chronic anxiety, and one of the reasons for maintaining this state is the patient’s persistent and debilitating focus on negative or potentially threatening life experiences (Robinson et al., 2013). The amygdala is a key brain region in the emotional regulation circuit. The study also found that, in addition to the amygdala, the anterior cingulate cortex (ACC) is another core brain region of concern in the emotional regulation of SA (Frick et al., 2018). The anterior cingulate cortex (ACC) also plays a crucial role in regulating emotions. It involves explicitly dealing with social rejection and coping with stress caused by social interactions (Klumpp et al., 2012; Schmid et al., 2015). Studies in the meta-analysis found that the activation of the left anterior cingulate gyrus was significantly reduced in SA patients compared to healthy controls (Yu et al., 2021). In a simple emotion recognition task, the researchers found an interaction of whole-brain amygdala connections within the anterior cingulate cortex cluster, which was caused by a significant increase in circuit coupling when patients processed fearful faces versus happy faces. Notably, this is consistent with contemporary psychiatric theory that circuit coupling is positively associated with self-reported anxiety symptoms, providing evidence for a persistent association between this circuit and subjective symptoms (Robinson et al., 2014).

(4) The method of treatment includes drug options, psychotherapy, and neuroregulatory interventions such as tDCS, which have been found to alter brain function (Frick et al., 2020; Li et al., 2016). Some researchers used brain signals as neural predictors of treatment outcomes in SA. Selective Serotonin Reuptake Inhibitor (SSRI) is the most commonly used medication for SA (Rappaport et al., 2021). Clinical and preclinical evidence suggests that SSRI is involved in the pathogenesis of SA (Arad et al., 2023). Research has found that 5-HT levels in the basal amygdala (BA) decrease during anxious states and increase during social activities (Yu et al., 2022). Other studies have shown that 5-HT promotes social behavior by affecting different brain regions (Wu et al., 2021). One study also examined the effects of selective serotonin reuptake inhibitors (SSRIs) on brain function in patients with OCD, PTSD, and SA, and found that patients with SA, OCD, and PTSD experienced reduced activity in the anterior cingulate gyrus, right thalamic cingulate gyrus, and left hippocampus after SSRI treatment (Bandelow et al., 2012). The study conducted by Azriel et al. (2024) further confirmed that SSRIs are both effective treatments for SA and lead to increased activation of the right inferior frontal gyrus and anterior cingular cortex during implicit social threat processing. These brain regions may be the neurobiological basis of SSRI efficacy. This suggests that SSRIs can have an impact on brain function in people with psychiatric disorders such as SA (Bandelow et al., 2012). This suggests that abnormal neural activity can be reversed by selective serotonin reuptake inhibitor treatment, and the activity in these brain regions may also serve as potential targets for predicting the efficacy of selective serotonin reuptake inhibitors.

Among psychotherapy, cognitive behavioral therapy (CBT) is the most well-studied non-pharmacological approach to treating SA, and its effectiveness has been demonstrated in numerous studies (Kindred et al., 2022). CBT is a time-bound and present-oriented psychotherapy approach that teaches patients the cognitive and behavioral skills needed to function adaptively in both interpersonal and intrapersonal contexts (Bemmer et al., 2021). CBT is considered the standard treatment for SA, and the study of the neuro predictors of its efficacy is conducive to personalized treatment. Preliminary evidence suggests that the higher visual cortex, dorsal anterior cingulate gyrus, medial/lateral dorsal prefrontal cortex, and orbitofrontal cortex are potential predictors of functional activation in brain areas related to cognitive control before intervention. Meanwhile, the amygdala is structurally and functionally connected with brain areas related to emotional regulation. Additionally, ERP components induced by emotional stimulation are associated with symptom improvement after treatment (Frick et al., 2020; Jiang et al., 2024; Sandman et al., 2020; Young et al., 2019). For example, right amygdaloid-right ventrolateral prefrontal cortex (vlPFC) connectivity was found to be a predictor of treatment response(Jafari et al., 2021). Decreased amygdaloid-DACC connectivity predicts the long-term efficacy of iCBT for SA (Månsson et al., 2013). Recent long-term studies have also confirmed that CBT for SA is effective in reducing SA symptoms for 12 months or more after stopping treatment (Kindred et al., 2022). The researchers also found that increased activity in the right prefrontal cortex and right middle occipital gyrus, along with decreased activity in the left posterior superior temporal gyrus, was associated with reduced symptoms of SA in individuals. This association was observed during the reevaluation of social criticism when comparing pre-CBT and post-CBT brain activity. This suggests that the potential effect of CBT changes the individual’s brain circuitry (Goldin et al., 2021). The findings could potentially be used to target brain circuits associated with clinical improvement to improve treatment effectiveness. It also suggests that neuroimaging can be used to predict treatment outcomes in patients with SA (Picó-Pérez et al., 2023).

In addition, neuroregulation has been emphasized as a form of treatment for a variety of conditions, including those with SA symptoms (Vergallito et al., 2021). tDCS is a viable treatment option for high-prevalence neuropsychiatric disorders and is important for understanding pathological and neuropsychological adaptation processes (Mohammadabadi et al., 2021). Dysfunction of the amygdala-frontal network is the core of SA pathophysiology, and the reaction process is decreased activity of the lateral prefrontal cortex (PFC) and hypersensitivity of the medial PFC and amygdala (Mizzi et al., 2022). The study found that modulating lateral-medial PFC activity through intensive stimulation can improve cognitive control, motivation, and emotional networks in SA patients, resulting in therapeutic effects (Jafari et al., 2021). Previous studies have linked anxiety to low activation of the left DLPFC, resulting in an inability to suppress the amygdala, making it overly involved in neural activity for threat detection and processing (Etkin and Wager, 2007). On the other hand, there is evidence that the right side DLPFC is more activated in anxiety disorders (White et al., 2023). This can lead to the emergence and chronicity of cognitive/emotional deficits (i.e., exaggerated fear response/threat perception). In a tDCS study, researchers used excitatory stimulation of the left prefrontal cortex and inhibitory stimulation of the right prefrontal cortex to reduce the severity of anxiety symptoms. The results produced by this method validate previous studies of tDCS and create an innovative model for up-down regulation mechanisms, which may serve as a guide for future systematic studies in this area (Vicario et al., 2019).

### 3.3 Research trend analysis

Analyzing the development trends of a research field helps scholars track cutting-edge topics and predict research trends, thereby guiding their research direction. In the field of visual studies, keyword burst maps and keyword co-occurrence timezone maps can help identify future research trends (Jamieson et al., 2023).

#### 3.3.1 Keyword burst analysis

The burst of keywords refers to a sudden increase in the number of occurrences of a keyword within a specific period, making it suitable for characterizing the research frontier and development trends in that field. This map not only illustrates changes in research focus but also highlights the most enduring and popular core keywords, helping researchers identify and understand the hotspots and their contexts within the field.

**FIGURE 7:**
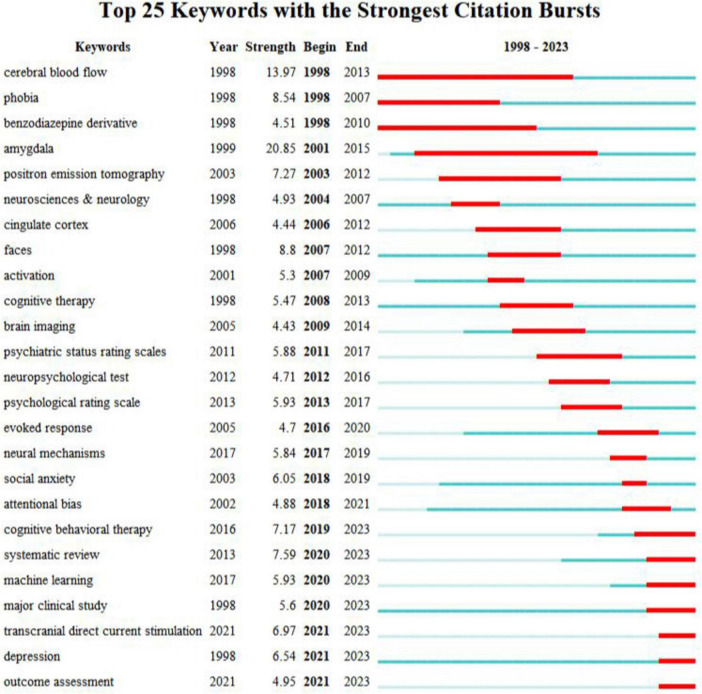
Top 25 keywords with the strongest citation burst.

As shown in Figure 7 using 2023 as the cutoff time for burst analysis reveals that the keywords with significant growth in recent years are “cognitive behavioral therapy,” “systematic review,” “machine learning,” “major clinical study,” “transcranial direct current stimulation,” “depression,” and “outcome assessment.” The respective burst strengths are 7.17, 7.59, 5.93, 5.6, 6.97, 6.54, and 4.95. These keywords are expected to appear frequently in the coming years and will become trends in research within this field.

Cognitive behavioral therapy (CBT) is an effective intervention for the treatment of SA, and the neuro markers to evaluate the treatment effect are at the forefront of research (Kindred et al., 2022; Su-Shou et al., 2021). Although existing studies have shown that CBT can improve anxiety symptoms, differences in the association of various neural markers with treatment response have been identified. This finding provides a new perspective for understanding the treatment mechanism and enhancing treatment outcomes (Frick et al., 2020; Sandman et al., 2020). Studies have shown that there are powerful neural predictors of CBT outcomes for anxiety-related disorders. Ultimately, these predictors can be combined with other data to develop a tailored treatment approach for these mental disorders (Picó-Pérez et al., 2023). In the future, it is necessary to combine multiple data sources and adopt big data and multimodal methods to more comprehensively identify the risk factors associated with SA treatment outcomes to improve treatment outcomes (Khosravi et al., 2022). CBT is an important way to treat anxiety and depression, and clinical practice can use the brain mechanism to see the treatment changes of patients more objectively, and then promote the following treatment plan. At the same time, CBT can also be co-treated with mindfulness to bring a better prognosis (Goldin et al., 2021).

At the same time, in the face of similar diseases, there is an urgent need for clinical treatment to derive quantitative measures based on coherent neurobiological dysfunction or “biotype.” Using the study of brain mechanisms, by designing different brain region activation tasks, it is possible to determine the type of disease in patients before clinical diagnosis. The results of brain activation provide a new, theoretically driven, clinically validated, and explainable quantitative approach to resolving the biological heterogeneity of depression and anxiety, representing a promising approach that can advance precision clinical care in psychiatry (Tozzi et al., 2024).

A systematic review, especially meta-analysis, was used to summarize existing research on brain function and brain structure in SA and to identify abnormal brain regions. Through the meta-analysis of different paradigms, we can further understand the neural mechanism of SA (Deng et al., 2023). Moreover, some meta-analytic studies have differentiated SA from other disorders, such as generalized anxiety disorder, post-traumatic stress disorder, specific phobia, and depression, revealing characteristic activation patterns in the brain (Liang et al., 2023; Pierce and Black, 2023; Yu et al., 2021). This approach helps uncover the neurobiological underpinnings of SA and provides a basis for identifying reliable biological markers, which has become a trend in recent years (Deng et al., 2023). In recent years, neurobiological research has primarily focused on comparing the mechanisms underlying anxiety with those of other disorders (Chavanne and Robinson, 2021; Schaffner, 2020). As well as using ALE meta-analysis to investigate the consistency of activation patterns elicited by anxiety (Yu et al., 2021). It can be inferred that examining the consistency and heterogeneity of anxiety pathogenesis using multidimensional modeling approaches, in comparison to other disorders, is a trend for future research.

The use of computers to assist in the treatment of mental illnesses aims to achieve this through interdisciplinary approaches that combine various aspects of psychiatry, neuroscience, mathematics, and artificial intelligence. By analyzing how changes related to diseases affect behavior at different levels of brain structure, diseases can be distinguished and explained (Khaleghi et al., 2022). Among them, machine learning (ML) techniques have been applied to SA neuroimaging research data to identify important neurobiological predictors of psychopathology (Al-Ezzi et al., 2020; Rezaei et al., 2023). ML is an advanced data analysis method that has the potential to enhance clinical decision-making, such as diagnostic evaluation. It can provide evidence-based insights to identify which brain regions exhibit the most significant differences between individuals with different diseases. (Pereira et al., 2009). One study showed that a machine learning model with resting-state brain functional radionics features has been successful in predicting SA levels in young adult participants by training them (Kim et al., 2022). Other researchers have used the neural correlations of threatening social signals to perform support vector machine analysis to classify SA (Xing et al., 2020). It was found that the support vector machine (SVM) of the brain’s response to the threat surface is a promising method for classifying SA. This method requires recording whole-brain activity on threatening or happy faces for optimal classification performance. Brain regions outside the fear circuit (e.g., amygdala) that appear relatively important in the classification highlight brain regions that contribute to the diagnosis of SA. Al-Ezzi et al. (2020) found that graph-theoretic network features combined with PDC are effective tools for SA identification, using SVMs to achieve maximum classification performance with accuracy (92.78%), sensitivity (95.25%), and specificity (94.12%). Tian et al. (2024) also studied the effect of EEG signals on SA induced by facial expression processing under the psychological paradigm from the perspective of deep learning. The results show that the ERP features show better anxiety classification performance than other features, especially the late positive potential, and show the advantages of stability and high accuracy. These findings provide new insights into the field of SA testing and help advance the development of its objective identification methods (Tian et al., 2024). In psychiatry, data-driven ML methods can be effectively used to predict treatment response outcomes (Chen et al., 2023). ML methods process large amounts of data to stratify patients based on specific clinical phenotypes for individualized treatment. To date, accuracy values exceeding 50% are considered clinically acceptable (Dadi et al., 2021). Emerging evidence highlights the potential benefits of machine learning approaches in the field of depression, including the application of diagnostic and personalized treatment strategies (Aleem et al., 2022). ML is expected to address the significant variability observed in treatment outcomes in the SA domain by providing a method for objectively assessing heterogeneous data.

Non-invasive neurostimulation, as a “non-invasive, economical, safe, and easy to operate” non-invasive physical therapy method, is increasingly favored in the clinical treatment of mental psychology and has become a research hotspot in the field of neuropsychiatric diseases (Jafari et al., 2021). The use of transcranial stimulation techniques, including transcranial magnetic stimulation, transcranial direct current stimulation, and transcranial photon biomodulation, has gained increasing attention for the intervention of anxiety disorders. Transcranial direct current stimulation (tDCS) is considered an effective method for treating anxiety disorders and fear responses (Mohammadabadi et al., 2021; Sousa et al., 2021). The core of the physiological and pathological mechanisms of SA lies in the dysfunction of the amygdala-prefrontal cortex network, characterized by insufficient activity in the dorsal prefrontal cortex (PFC) and hypersensitivity in the medial PFC and amygdala. Additionally, the amygdala is functionally modulated by both cortical and subcortical structures. Stimulating the dorsolateral prefrontal cortex to alter the activity of the prefrontal-amygdala pathway can effectively regulate the attention of anxious individuals to fear-inducing stimuli (Ironside et al., 2019; Jafari et al., 2021) tDCS can achieve non-invasive and precise neural circuit regulation of cortical targets (D’Onofrio et al., 2023). For example, some studies use tDCS to modulate dorsolateral and medial PFC activity to reduce the core symptoms of SA and attention bias in response to threatening stimuli (Jafari et al., 2021). These findings provide important theoretical guidance and experimental evidence for improving individual clinical anxiety disorders and predicting post-intervention efficacy by precisely targeting cortical targets (George et al., 2009). tDCS has broad application prospects in the treatment of anxiety, mood, cognitive impairment, etc.

“Major clinical study” and “Outcome assessment” are also a keyword that has proliferated in recent years, which also suggests that on the one hand, although advanced and multivariate neuroimaging techniques have been used to probe the neural mechanisms and core brain regions of SA, the results still need a large number of clinical studies to evaluate the research results (Groenewold et al., 2023). On the other hand, it is also important to improve the evaluation of the effect of treatment plans through objective “outcome assessment” to promote the design and implementation of “major clinical study.” In recent years, the use of fMRI and ERP to study the neural markers of treatment effects in SA and to predict treatment outcomes has become a research hotspot (Jiang et al., 2024; Picó-Pérez et al., 2023). It was found that the variability of transient fMRI signals may be a highly reliable and valid prognostic indicator for the clinical prognosis of SA (Månsson et al., 2022); visual mismatch negativity (vMMN) is also a neuro marker of the clinical treatment effect of SA, which is a neuro marker reflecting the clinical efficacy of ABM in SA (Arad et al., 2019).

#### 3.3.2 Time zone map analysis

**FIGURE 8:**
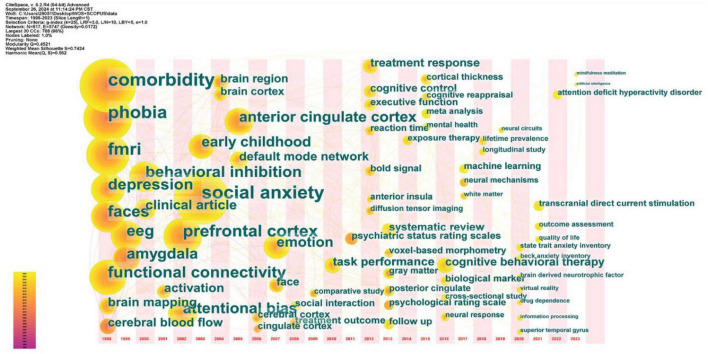
The keyword time zone map about research hotspots and trends of neuroimaging in SA.

The time zone map analysis can visualize the evolution and development trend of keywords in different periods. In CiteSpace, we select “keyword” as a node, and use time zone view to help us better understand and analyze the evolution of hot topics in the field of SA brain mechanism, and observe the emergence of new keywords over time. The location of the nodes in the graph represents the year of the first discovery of the keywords, and the greater the number and richer the color of the lines formed between the nodes, the more active the keywords are in the related scientific research application fields. In this paper, we select the literature from 1998 to 2023 and present the time zone graph with 1 year as a phase (Figure 8).

By 2003, Research began using emotional paradigms such as faces to explore the facial processing mechanisms of SA, with fMRI and PET being the primary tools for studying the internal brain mechanisms of the disease. The research objectives and methods were relatively clear and focused, with some topics receiving in-depth exploration and investigation, as seen in the early stages of Figure 8 with terms like “comorbidity,” “phobia,” “faces,” “emotion,” “fMRI,” “amygdala,” “depression” etc.

After 2003, with the increased focus on the field of SA, the frequency of use of the term “SA” continued to rise, and the scope of research became more diversified. In addition to focusing on faces and emotions, Researchers paid more attention to the study of brain regions interested in this disease, the functional connections between different parts of the brain, and the exploration of comorbidity mechanisms with SA, such as the terms “attentional bias,” anterior cingulate cortex,” and “functional connectivity” found in the middle section of Figure 8.

Since 2012, research into the neural mechanisms of SA has been in an explosive state, with comprehensive and specific studies. The research includes using machine learning methods for the classification and diagnosis of mental disorders; exploring the mechanisms of cognitive-behavioral therapy, exposure therapy, virtual reality, and tDCS as neuro-regulatory treatments; revealing the neural mechanisms of cognitive dysfunction, such as executive control and cognitive reappraisal; comparing the neural mechanisms of co-morbidity with ADHD; and summarizing the research findings using meta-analysis and systematic literature review methods.

In general, the research process of SA has undergone an evolution from a single study to a multidisciplinary approach, from simple methods to high-tech methods, and from superficial phenomena to deep mechanisms. In recent years, researchers have begun to try to construct models to better understand and explain the brain mechanisms of SA, which indicates a possible direction for future SA research.

## 4 Limitations and future research

However, there are also some limitations to our study. Since the literature screening criteria in this study were limited to English, some important literature in other languages may have been omitted. Therefore, the results of this study should be carefully extrapolated to countries with different languages. For example, cultural and individual differences need to be taken into account, particularly the role of the collective orientation of Eastern cultures versus the individualistic orientation of Western cultures, which will help deepen and refine our understanding of SA.

Combined with the development track of SA research and the analysis of CiteSpace, we analyze future research and development trends in the field of SA brain mechanisms. Future research directions include the following:

Firstly, expanding the research population to reveal the developmental and changing patterns of SA is necessary. According to our bibliometric analysis, we found that the subjects in the field of SA brain mechanisms are often relatively young individuals. This may be due to factors such as the early onset of the disease and the fact that younger individuals are in a developmental stage, leading most researchers to choose children and adolescents as their subjects. Considering that individuals of different ages may experience varying degrees and causes of anxiety, age and physiological factors could also be reasons for some divergent research findings. However, psychological research should serve the entire society, and given that the lifetime prevalence of SA ranges between 8.4 and 15% with a high rate of comorbidity with other disorders (Weise et al., 2024), it is essential to broaden the scope of research in the SA field. We should not be limited to childhood, adolescence, and early adulthood, patients in middle adulthood and seniors also need attention.

Secondly, employing multimodal technologies is crucial to fully uncover the brain mechanisms of SA. Meanwhile, neurobiological models of SA were constructed based on brain imaging research findings. Due to the complexity of the causes of SA and the incomplete uniformity of research conclusions, researchers need to explore its pathogenesis from multiple perspectives so that cross-cultural and multimodal technologies will become the general trend in research. Currently, research into the brain mechanisms of SA primarily utilizes various brain imaging techniques independently, especially magnetic resonance imaging (MRI). These studies investigate changes in brain structure among patients and various control groups across different cognitive and emotional task paradigms (Al-Ezzi et al., 2020; Bas-Hoogendam et al., 2020; Poole et al., 2021). However, given the diverse characteristics of SA patients and the similarities with other diseases, it is necessary to conduct examinations from multiple dimensions, such as physiological and behavioral aspects, and even through the analysis of audio and video data (Weise et al., 2024). Among these, functional near-infrared spectroscopy (fNIRS) has a better temporal resolution than fMRI and offers superior spatial resolution compared to EEG, making it suitable for use in conjunction with these techniques, depending on the experimental objectives. Future researchers should integrate multimodal data to build predictive models for the classification and diagnosis of SA. This contributes to a deeper understanding and prediction of the pathogenesis of SA (Chavanne and Robinson, 2021; Jamieson et al., 2023). Despite the substantial evidence from psychological and clinical perspectives supporting cognitive models of SA (Nd et al., 2020; Neumann et al., 2010), data from neurobiology research has been scarce. Technological advances in genetics, biology, and functional neuroimaging have enabled researchers to demonstrate that genetic and biological factors and their impact on neural function play a significant role in the onset and maintenance of SA (Bas-Hoogendam et al., 2016, 2020; Carvalho et al., 2020). Numerous studies have provided a framework for the relationship between cognitive processes, neural dysregulation, emotional disturbances, social context, and SA (Hofmann et al., 2012; Neumann et al., 2010; Schaffner, 2020). These research advancements have significantly enhanced our understanding of the relationship between cognitive, biological, and social factors in the pathogenesis of SA. They have also achieved breakthrough progress. Genetic and neurobiological studies have elucidated some potential pathogenic pathways for SA and suggested the biological relevance of cognitive models to SA, which have significant practical implications (Frick et al., 2020).

Thirdly, employing hyperscanning brain imaging technology to investigate the characteristics of brain activity during real social interactions can enhance the ecological validity of research (Carollo and Esposito, 2024; Saul et al., 2022). One of the primary barriers to SA is difficulty in interpersonal communication, and hypers-canning is an effective technique for examining neural activity synchronization and information flow between brains (Saul et al., 2022). However, existing studies have participants lying inside an fMRI machine imagining interaction scenarios, which fails to observe the brain’s interactive characteristics of SA in natural interaction contexts. This suggests that the choice of study design and research method needs to be coordinated with the choice of instrument. In recent years, interbrain cognitive synchrony has become a hot topic (Al-Ezzi et al., 2020), which could facilitate research on the brain activity of socially anxious individuals during social interactions, thereby improving the external validity and applied value of the research (Deng et al., 2024; Konrad et al., 2024).

Fourth, there is a need to strengthen research on non-invasive brain stimulation interventions such as tDCS. Although existing neurofeedback technologies have identified several effective EEG targets for treating anxiety disorders and are gradually being used in clinical interventions, current targets mostly focus on neural oscillations in localized brain regions (Ferrarelli and Phillips, 2021). There is a lack of research targeting neural circuit-level biomarkers. As the neural circuits associated with SA are gradually uncovered, it becomes possible to modulate these circuits through neurofeedback techniques to intervene in anxiety disorders, and there is already research confirming this (Paes et al., 2013). By examining the modulatory effects of neurofeedback training on neural circuits and its alleviating effects on anxiety disorders, we aim to determine the effectiveness of new targets and ultimately develop active neuromodulation techniques that can effectively alleviate anxiety.

In conclusion, future research should conduct in-depth studies on the brain mechanisms of SA by applying multimodal techniques systematically. Meanwhile, machine learning methods should be utilized to construct neural prediction models, addressing the challenges of comorbidity in the clinical diagnosis of SA, enhancing the accuracy of clinical diagnosis of SA, precisely predicting individual differences in the treatment effects of SA, providing objective neurobiological markers for the diagnosis and treatment response of SA, and facilitating differentiated clinical decision-making.

## 5 Conclusion

This study quantitatively analyzed the publication volume, journals, cited references, authors, institutions, and keywords in the field of brain mechanisms of SA from 1998 to 2023. It aims to help researchers get a clear picture of the research overview, hotspots, and trends in the field. The main findings are as follows:

(1) From the current analysis of the brain mechanisms of SA, the number of publications has been steadily increasing. Most authors collaborate closely. Among them, Pine’s team, which published a total of 59 articles, is also the most central. There are 3 institutions with more than 50 publications each, and Harvard University has the highest centrality. At present, This field has significant development prospects for the future. But at the same time, the correlation between article frequency and centrality and the brain mechanism of SA is not linear, that is, the wider the scope of the study design, the more attention it receives. This suggests that we need to consider both centrality and relevance when selecting core literature for reference.
(2) Based on keyword co-occurrence and cluster analysis, the research hotspots in this field can be categorized into research techniques, research areas, core brain regions and brain networks, and the neural predictors of treatment outcome in SA. fMRI and ERP are widely used to explore the neural mechanisms of cognitive processing (such as face processing, attentional bias, and behavioral inhibition) in SA, as well as using brain signals as predictors of treatment outcomes in SA. tDCS as a neuromodulation technology has been used to explore the effectiveness and mechanism of treatment SA. Studies have suggested that a characteristic of SA patients is the dysfunction of the limbic-prefrontal circuit, particularly centering around the prefrontal cortex and amygdala, as well as DMN, which is the most studied network. In addition, exploring the comorbidity of SA and depression using brain imaging technology is also a hot research topic. Due to the complexity of the causes of SA and the incomplete uniformity of research conclusions, researchers need to explore its pathogenesis from multiple perspectives. Multimodal research can more comprehensively understand the complexity of SA, occupy a higher research perspective, and obtain more accurate and insightful research results.
(3) The analysis of the keyword burst map and time zone map shows the evolution results in the time dimension. This change in research focus is that as researchers continue to ask about the results, each node is a breakthrough in the field of research on the brain mechanism of SA. Currently, there is a shift toward investigating the abnormalities in functional connectivity patterns among individuals with SA, conducting meta-analyses to identify abnormal brain regions in this field, and consequently building models to explore the neural mechanisms underlying SA. The future research trend in SA brain mechanisms will mainly focus on the precise prediction of SA onset and using meta-analysis and modeling to detect neurobiological markers of SA, constructing neurobiological models of SA based on brain imaging research findings as well as using brain signals as predictors of treatment outcomes in SA, aiming to identify the neural mechanisms of SA and ensure more accurate clinical diagnosis associated with clinical improvement to improve treatment effectiveness.

Through this study, we analyzed the literature on the brain mechanisms of SA and found that the research focus in this field has evolved, giving readers a clear context. At the same time, based on the research results and the analysis of the current situation in this field, we discussed future research directions and challenging but urgent problems to be solved, hoping to provide new ideas and directions for readers and the study of the brain mechanism of SA.
